# DETERMINANTS OF QUALITY OF LIFE IN COMMUNITY-DWELLING OLD ADULTS

**DOI:** 10.1093/geroni/igz038.1792

**Published:** 2019-11-08

**Authors:** Alfons Ramel

**Affiliations:** University of Iceland, Reykjavik, Capital area, Iceland

## Abstract

Background: Quality of life (QoL) has been regarded as a critical predictor of successful aging in gerontological research. Aim: The aim of this study was to examine the associations between physical activity, muscle strength, body composition, physical-/cognitive function and disease with quality of life community-dwelling older adults. Methods: Participants (N=225, 73.7±5.7yrs, 58.2% female) from the Reykjavik capital area in Iceland took part in this cross-sectional study. Socioeconomics, QoL, body composition, muscular strength, timed up and go test (TUG), six minute walk for distance (6MWD) and disease related information were measured. Fasting blood samples were analyzed for routine clinical measures. Results: In our subjects, only 19.1% had QoL below the age and gender corrected norm score of 50. A simple comparison between subjects with QoL below 50 vs subjects with a score above 50 indicated that participants with higher QoL had higher physical and cognitive function, higher muscular strength, lower blood glucose, exercised more and used a lower number of medicines. Differences in education, smoking, alcohol consumption, dietary intake and gender distribution were not significant. According to age and gender corrected linear models, TUG (B=-0.54,P=0.022), number of drugs (B=-0.67,P=0.018) and fasting glucose (B=-0.96,P=0.025) were the strongest independent correlates of QoL. In the models insulin/glucose and TUG/6MWD were interchangeable. Conclusion: Physical function, number of drugs and glucose metabolism are independently related to QoL and represent therefore potentially modifyable targets for future interventions in order to improve QoL in community dwelling old adults.

